# Fourier domain optical coherence tomography to assess the iridocorneal angle and correlation study in a large Caucasian population

**DOI:** 10.1186/s12886-016-0219-z

**Published:** 2016-04-18

**Authors:** José Ignacio Fernández-Vigo, Julián García-Feijóo, José María Martínez-de-la-Casa, Javier García-Bella, Pedro Arriola-Villalobos, Cristina Fernández-Pérez, José Ángel Fernández-Vigo

**Affiliations:** Department of Ophthalmology, Hospital Universitario Clínico San Carlos, Instituto de Investigación sanitaria San Carlos (IdISSC), c/Profesor Martín Lagos s/n, 28100 Madrid, Spain; Centro Internacional de Oftalmología Avanzada, Madrid, Spain; Department of Preventive Medicine, Hospital Universitario Clínico San Carlos, Instituto de Investigación sanitaria San Carlos (IdISSC), Madrid, Spain; Department of Ophthalmology, Universidad de Extremadura, Badajoz, Spain

**Keywords:** Anterior chamber, Iridocorneal angle, Glaucoma, Optical coherence tomography

## Abstract

**Background:**

Recently, novel anatomic parameters that can be measured by optical coherence tomography (OCT), have been identified as a more objective and accurate method of defining the iridocorneal angle. The aim of the present study is to measure the iridocorneal angle by Fourier domain (FD) OCT and to identify correlations between angle measurements and subject factors in a large healthy Caucasian population.

**Methods:**

A cross sectional study was performed in 989 left eyes of 989 healthy subjects. The iridocorneal angle measurements: trabecular-iris angle (TIA), angle opening distance (AOD_500_) and trabecular-iris space area (TISA_500_) 500 μm from the scleral spur, were made using the FD-OCT RTVue®. Iris thickness was also measured. Correlations were examined between angle measurements and demographic and ocular factors. The main determinants of angle width were identified by multivariate linear regression.

**Results:**

TIA could be measured in 94 % of the eyes, and AOD_500_ and TISA_500_ in 92 %. The means recorded were TIA 35.8 ± 12.2 degrees (range 1.5 to 76.1), AOD_500_ 542.6 ± 285.4 μm (range 15 to 1755), and TISA_500_ 0.195 ± 0.104 mm^2^ (range 0.02 to 0.62). The correlation between the temporal and nasal quadrant was R = 0.902 for TIA. The reproducibility of measurements was excellent (intraclass correlation coefficient >0.947). Mean angle width measurements were smaller in women (p = 0.02). Correlation was detected between angle means and anterior chamber volume (ACV; R = 0.848), anterior chamber depth (ACD; R = 0.818), spherical error (R = -0.619) and age (R = -0.487), while no correlation was observed with Intraocular pressure (R = -0.052). ACV emerged as the main determinant of TIA (R^2^ = 0.705; *p* < 0.001).

**Conclusions:**

In this Caucasian population, strong correlation was detected between FD-OCT anterior angle measurements and ACV, ACD, spherical refractive error and sex, emerging the ACV as the main determinant of TIA.

## Background

Anterior segment optical coherence tomography (OCT) is a recent addition to the imaging techniques available such as devices based on Scheimpflug technology or Ultrasound biomicroscopy, to measure the anterior chamber angle, or iridocorneal angle, [1–6] as a way to identify subjects with occludable angles or those at risk of angle closure. OCT has evolved from time-domain to Fourier-domain (FD) or spectral-domain systems and now offers enhanced signal-to-noise ratio, image acquisition speed and resolution [7, 8].

Several subject factors have been classically linked to a risk of developing primary angle-closure glaucoma [9] such as female sex, hyperopia, advanced age and a narrow anterior chamber [10–12]. More recently, several novel anatomic parameters that can be measured by OCT, including trabecular-iris angle (TIA), angle opening distance (AOD) and trabecular-iris space area (TISA), [13] have been identified as a more objective and accurate method of defining the iridocorneal angle. These new parameters allow for a more accurate assessment of the factors linked to angle closure.

Most large studies performed to date on the iridocorneal angle have examined Asian subjects [10–12, 14–16]. However, there are scarce data available for Caucasians despite differences, as some studies have demonstrated that Chinese have a narrower anterior angle than Caucasian eyes, even after correcting for anterior chamber depth [17, 18].

The aim of the present study was to measure anterior angle width by FD-OCT in a large population of healthy Caucasian subjects and to examine possible correlations with demographic and ocular factors. The reproducibility of the angle measurements made was also assessed.

## Methods

A cross-sectional study was performed in 1006 healthy subjects consecutively recruited among patients visiting the Centro Internacional de Oftalmología Avanzada in Madrid (Spain) for a routine eye examination over the period November 1, 2012 to June 30, 2013. The study protocol adhered to the tenets of the Declaration of Helsinki and received Institutional Review Board approval from the above center. After undergoing a complete medical history and full eye examination, signed informed consent was obtained from subjects meeting the study’s inclusion and exclusion criteria.

### Subjects

Inclusion criteria were an age between 18 and 85 years and Caucasian ethnicity. Subjects were excluded if they had been diagnosed with an eye disease or if any eye disorder was detected in the exam such as glaucoma or a mature cataract, previous ocular surgery, a history of ocular trauma, or an ocular or iridocorneal angle abnormality. Also excluded from the study were subjects with a physical or mental condition that could hinder the examination, and subjects under treatment with any medication that could affect intraocular pressure (IOP).

### General examination protocol

All participants underwent a standard examination including general medical history, visual acuity, slit-lamp anterior biomicroscopy, posterior segment ophthalmoscopy and IOP measured using a Canon TX 10® pneumotonometer (Canon Inc.; Tokyo, Japan). Participants were also subjected to a Pentacam® (Oculus Inc.; Wetzlar, Germany), IOL Master® (Carl Zeiss, Meditec, USA) and OCT RTVue® (Optovue Inc., Fremont, CA, USA) examination on the same day. The room lighting conditions for all tests were set at 7 EV, or 320 lux, using a Flashmate K-308S® light meter (SEKONIC, Tokyo, Japan).

The eye randomly selected for all measurements was the left eye of each participant. The measurements made with the Pentacam® were anterior chamber volume (ACV) and depth (ACD) (measured from the epithelium) and central corneal thickness (CCT). The IOL Master® was used to measure axial length (AL), corneal diameter or white-to-white distance (WTW), and pupil diameter.

### Fourier Domain OCT

A Fourier domain OCT RTVue® 100 with a CAM-L lens was used for the anterior angle measurements and for iris thickness. Exams were performed on the nasal and temporal quadrants (3 and 9 o’clock) under mesopic conditions with the device’s software set to Angle mode. In this mode, a 3 × 2.3 mm area was subjected to 32 B-scans, each comprised of 1024 A-scans, centered at the limbus. Each B-scan lasted 0.04 seconds.

Images were obtained by a trained examiner (JIFV) with the patient sitting and looking ahead. Only images of a quality indicated by a signal strength intensity (SSI) greater than 30 were accepted. Each quadrant was scanned three times and the examiner chose the image showing the best quality and least noise.

**Fig. 1:**
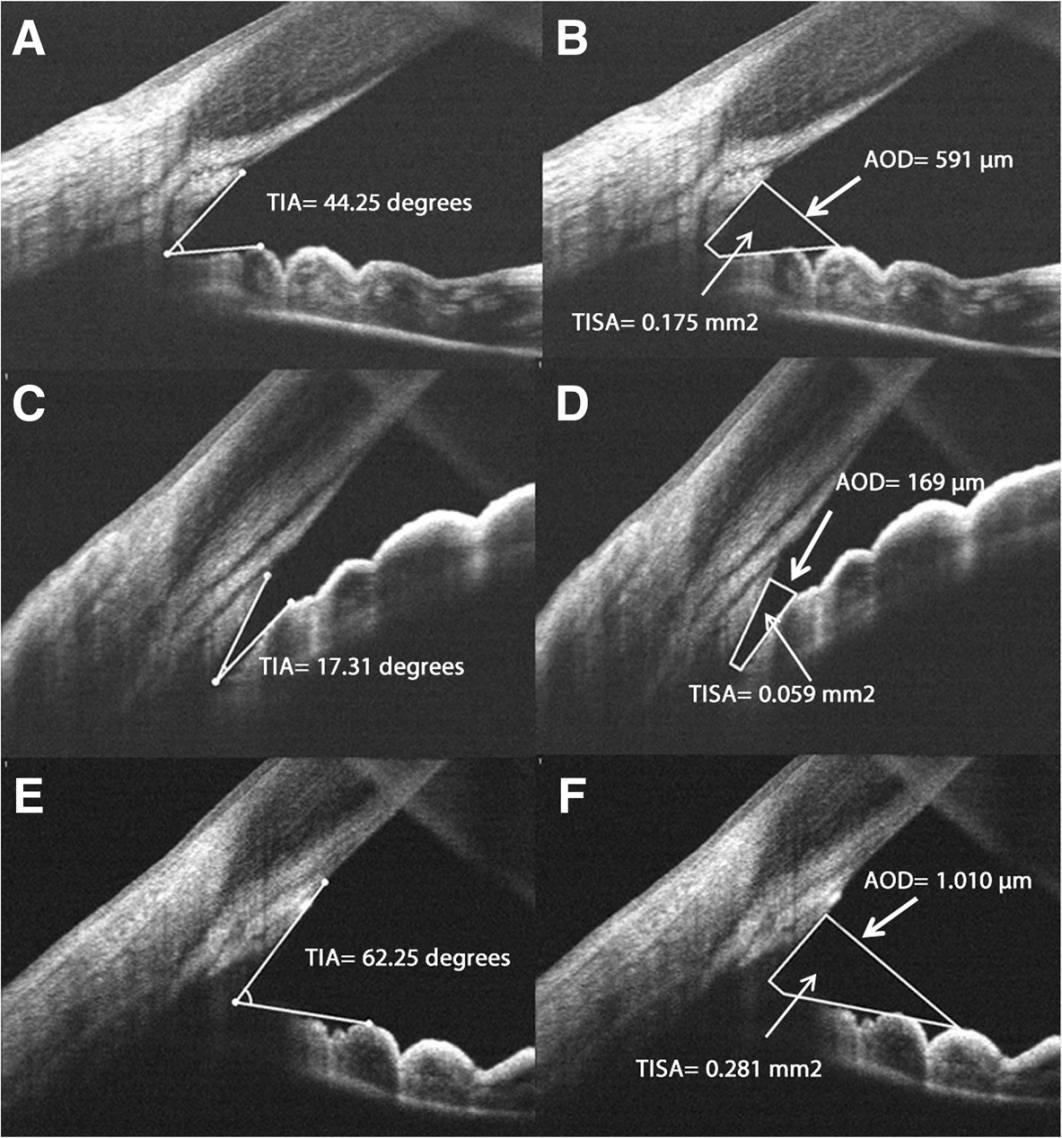
Examples of iridocorneal angle measurements made by Fourier Domain optical coherence tomography images. **a** Trabecular-iris angle (TIA) and (**b**) Angle opening distance 500 μm from the scleral spur (AOD_500_) and trabecular-iris space area (TISA_500_) 500 μm from the scleral spur in a normal angle width. **c** and **d** TIA, AOD_500_ and TISA_500_ measurements in a narrow angle. **e** and (**f**) TIA, AOD_500_ and TISA_500_ measurements in a wide angle

The measurements made manually on these images were trabecular-iris angle (TIA), angle opening distance 500 μm from the scleral spur (AOD_500_) and trabecular-iris space area (TISA_500_) 500 μm from the scleral spur [6, 11]. In Fig. 1, it may be seen how these measurements were made. TIA was measured by tracing a line from the angle recess to the Schwalbe’s line and another line on the surface of the iris to the perpendicular point on the Schwalbe’s line. AOD_500_ was measured as the perpendicular distance from the trabecular meshwork, 500 μm anteriorly from the scleral spur to the anterior iris surface. TISA_500_ was defined as the area bounded anteriorly by the AOD, posteriorly by a line drawn from the scleral spur perpendicular to the plane of the inner scleral wall to the opposing iris, superiorly by the inner corneoscleral wall, and inferiorly by the iris surface. Using this instrument, iris thickness was also measured manually as the perpendicular distance from the trabecular meshwork 500 μm anteriorly to the scleral spur (IT_500_).

### Reproducibility

To assess the reproducibility of angle measurements made using the RTVue® OCT, measurements were made in a subset of 50 patients selected at random from our study population. To determine interobserver reproducibility, angle measurements were independently made on the images obtained in the initial exam by two observers (JIFV and JAFV). One observer (JIFV) repeated the scanning with OCT and the angle measurements two weeks after the first examination to determine intraobserver reproducibility. In this subset, we also compared angle measurements between the right and left eyes.

### Statistical analysis

All statistical tests were performed using the software package SPSS® (Statistical Package for Social Sciences, v18.0; SPSS Inc., Chicago, IL). Quantitative data are provided as their means and standard deviations, and qualitative data as their frequency distributions. The Kolmogorov-Smirnov test was used to determine the distribution of the variables measured. Univariate correlations were established by Pearson correlation and multivariate correlations by linear regression as R^2^ values. For variables showing a non-normal distribution (spherical refractive error, AOD_500_ and TISA_500_) the Spearman’s Rho test was used. In addition a multivariate linear regression stepwise analysis calculating R^2^ was performed to identify the main factors determining a greater or lesser width of the iridocorneal angle. The reliability of the FD-OCT measurements was assessed by calculating intraclass correlation coefficients (ICC) for interobserver and intraobserver reproducibility. Significance was set at *p* < 0.05*.*

## Results

Iridotrabecular contact was observed in 17 eyes (1.6 %) with the FD-OCT. These eyes were excluded, because they could cause secondary alterations to the angle, being finally studied a sample of 989 eyes of 989 subjects.

Mean subject age was 49.1 ± 15.2 years (range 18 to 84); 615 were women (62 %). Mean intraocular pressure was 15.8 ± 3.5 mmHg (range 6 to 25) and the mean spherical error was -0.40 ± 3.55 diopters (range -14 to 8.25). The remaining variables examined are shown in Table 1.

**Table 1:** Measurements made in the study population using the Pentacam and the IOL Master

Ocular characteristics of the study subjects						
	Pentacam®			IOL Master®		
	ACD (mm)	CCT (μm)	ACV (mm^3^)	Axial length (mm)	WTW (mm)	Pupil diameter (mm)
Mean	3.35	550.6	161.8	23.87	12.11	4.26
SD	0.43	31.9	48.0	1.54	0.41	0.98
Maximum	4.61	643	265	33.61	13.40	6.45
Minimum	2.16	474	67	20.02	10.90	2.10

*ACD* anterior chamber depth, *CCT* central corneal thickness, *ACV* anterior chamber volume, *WTW* white-to-white or corneal diameter, *SD* standard deviation

TIA could be measured in 939 eyes (94.9 %) and averaged at 35.8 ± 12.2 degrees (range 1.5 to 76.1) in the temporal quadrant, and could be measured in 931 eyes (94.1 %) in the nasal quadrant and averaged 35.7 ± 12.3 degrees (range 2.2 to 74.8). Mean AOD_500_ was measured in 922 and 920 eyes (93.2 and 93.0 %) in the temporal and nasal quadrants respectively, being the means 542.6 ± 285.4 μm (range 15 to 1755) and 541.9 ± 284.8 μm (range 19 to 1741) respectively. TISA_500_ were 0.195 ± 0.103 mm^2^ (range 0.02 to 0.62 mm^2^) and 0.193 ± 0.101 mm^2^ (range 0.02 to 0.60 mm^2^) in the temporal and nasal quadrant respectively. IT_500_ was 395.5 ± 71.7 μm (range 194 to 910) in the temporal quadrant and 398.1 ± 73.2 μm (range 198 to 921) in the nasal one. All four variables were normally distributed.

The intra- and interobserver reproducibility observed for the angle measurements (TIA, AOD_500_ and TISA_500_) were ICC >0.962 and >0.947, respectively (Table 2).

**Table 2:** Intra- and interobserver reproducibility of anterior chamber angle measurements made by Fourier Domain OCT

Intraobserver reproducibility	Mean ± SD	ICC	95 % CI	*P* value
TIA Session 1 (degrees)	33.66 ± 11.38	0.979	0.969–0.986	<0.001
TIA Session 2 (degrees)	33.69 ± 10.94			
AOD_500_ Session 1 (μm)	491.16 ± 281.25	0.991	0.987–0.994	<0.001
AOD_500_ Session 2 (μm)	492.02 ± 280.84			
TISA_500_ Session 1 (mm^2^)	0.178 ± 0.104	0.962	0.944–0.974	<0.001
TISA_500_ Session 2 (mm^2^)	0.181 ± 0.112			
Interobserver reproducibility	Mean ± SD	ICC	95 % CI	*P* value
TIA Observer 1 (degrees)	33.66 ± 11.38	0.954	0.933–0.969	<0.001
TIA Observer 2 (degrees)	33.03 ± 10.55			
AOD_500_ Observer 1 (μm)	491.16 ± 281.25	0.975	0.964–0.983	<0.001
AOD_500_ Observer 2 (μm)	480.15 ± 295.72			
TISA_500_ Observer 1 (mm^2^)	0.178 ± 0.104	0.947	0.922–0.964	<0.001
TISA_500_ Observer 2 (mm^2^)	0.174 ± 0.116			

*TIA* trabecular-iris angle, *AOD*_*500*_ angle opening distance 500 μm from the scleral spur, *TISA*_*500*_ trabecular-iris space area 500 μm from the scleral spur. *ICC* intraclass correlation coefficient, *95 % CI* = 95 % confidence interval

The correlation between the temporal and nasal quadrant were R = 0.902 for TIA, R = 0.906 for AOD_500_, and R = 0.915 for TISA_500_ (both, *p* < 0.001).

TIA was 4.49 degrees greater in men than in women (95 % CI 3.35 to 5.64 degrees; *p* < 0.001), being the means 38.60 and 34.11 degrees respectively.

**Fig. 2:**
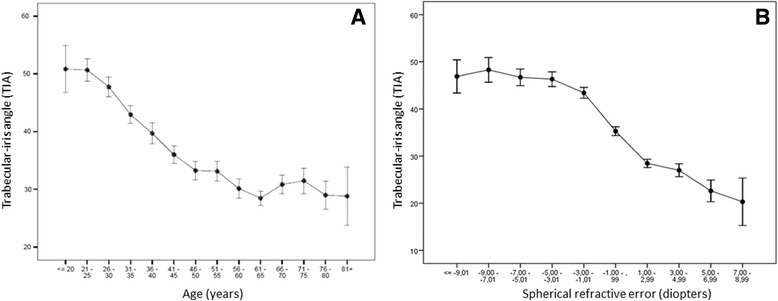
**a** Relationship between trabecular-iris angle (TIA) and age. Angle width measurements diminished from 18 years to 56–60 years, and stabilized thereafter. **b** Relationship between TIA and spherical refractive error. Mean trabecular-iris angle (TIA) was stable over the range < -9 to -3 diopters, point from which it was decreasing progressively toward hyperopia

Univariate correlation was observed between age and TIA (R = -0.487; *p* < 0.001). Angle width measurements diminished from 18 years to 56–60 years, and stabilized thereafter (Fig. 2). Correlation was observed in subjects ≤60 years (R = -0.496; *p* < 0.001) but not >60 years (R = 0.015; *p* < 0.001). The linear regression model identified a change in TIA per year until the age of 60 years (B = -0.579; 95 % CI -0.632 to -0.526; *p* < 0.0001).

Overall correlation between TIA and spherical error was R = -0.619 (*p* < 0.001). Mean TIA was stable over the range < -9 to -3 diopters, from which point it decreased until 20.31 degrees in hyperopes of >7 diopters (Fig. 2). For each diopter increase in spherical error from -3 diopters to positive values, angle width variation was B = -2.678 (95 % CI -2.906 to -2.450; *p* < 0.001).

No correlation was observed between TIA and IOP whether all eyes were considered (R = -0.052; *p* = 0.001) or only those with narrow angles (R = -0.112; *p* = 0.045; angle ≤20 degrees, *n* = 101 eyes). The factor showing greatest correlation with TIA was ACV followed by ACD (R = 0.848 and R = 0.818 respectively; *p* < 0.001). Table 3 shows the correlations observed between the angle measurements and the remaining subject factors examined. The average intra-subject right eye/left eye correlation was R = 0.886 (*p* < 0.001) for TIA, R = 0.880 (*p* < 0.001) for AOD_500_ and R = 0.870 (*p* < 0.001) for TISA_500_.

**Table 3:** Correlations between the angle parameters (TIA, AOD_500_ and TISA_500_) and different factors of the subjects

Demographic and ocular correlations with TIA, AOD_500_ and TISA_500_			
Factor	TIA	AOD_500_	TISA_500_
Age	-0.496 (*p* < 0.001)	-0.484 (*p* < 0.001)	-0.454 (*p* < 0.001)
ACD	-0.818 (*p* < 0.001)	0.760 (*p* < 0.001)	0.732 (*p* < 0.001)
IOP	-0.052 (*p* < 0.001)	-0.069 (*p* = 0.038)	-0.076 (*p* < 0.001)
CCT	-0.095 (*p* < 0.001)	-0.092 (*p* = 0.006)	-0.086 (*p* < 0.001)
ACV	0.848 (*p* < 0.001)	0.802 (*p* < 0.001)	0.769 (*p* < 0.001)
Pupil size	0.179 (*p* < 0.001)	0.187 (*p* < 0.001)	0.159 (*p* < 0.001)
WTW	0.269 (*p* < 0.001)	0.228 (*p* < 0.001)	0.216 (*p* < 0.001)
Axial length	0.599 (*p* < 0.001)	0.570 (*p* < 0.001)	0.562 (*p* < 0.001)
Spherical error	-0.575 (*p* < 0.001)	-0.545 (*p* < 0.001)	-0.540 (*p* < 0.001)
Iris thickness	0.151 (*p* < 0.001)	0.188 (*p* < 0.001)	0.208 (*p* < 0.001)

*TIA* trabecular-iris angle, *AOD*_*500*_ angle opening distance 500 μm from the scleral spur, *TISA*_*500*_ trabecular-iris space area 500 μm from the scleral spur, *ACD* anterior chamber depth, *IOP* intraocular pressure, *CCT* central corneal thickness, *ACV* anterior chamber volume, *WTW* white to white or corneal diameter

Eight variables were included in the multivariate regression model constructed to examine the effects on final TIA variability. Age and AL were excluded by the model, to give an optimal model containing the variables: ACV, ACD, sex, spherical error, IT_500_ and WTW that could explain 76.4 % of the variation in TIA (R^2^ = 0.764; *p* < 0.001). When these variables were individually analyzed, ACV (adjusted R^2^ = 0.705) emerged as the main model determinant, followed by ACD (adjusted R^2^ = 0.658), spherical error (adjusted R^2^ = 0.256), WTW (adjusted R^2^ = 0.057), sex (adjusted R^2^ = 0.029), and IT_500_ (adjusted R^2^ = 0.019).

## Discussion

This study conducted in a large population of healthy Caucasians examines iridocorneal angle width and its correlations with several subject factors. Mean TIA in our subjects was 35.8 ± 12.2 degrees in the temporal quadrant, and 35.7 ± 12.3 degrees in the nasal one. This value is fairly consistent with the values of 35.3 ± 8.5 degrees obtained using the RTVue OCT and 35.5 ± 9 degrees with the OCT Visante by Wylegala et al. [7] and of 38.3 ± 16.3 degrees using the slit lamp OCT by Xu et al. [10]. Mean AOD_500_ and TISA_500_ in our study population were 542.6 ± 285.4 μm and 541.9 ± 284.8 μm; and 0.195 ± 0.103 mm^2^ and 0.193 ± 0.101 mm^2^ in the temporal and nasal quadrant respectively. Reported figures for these variables vary widely due to the different populations examined including those of Leung [19] (572 ± 275 μm and 0.193 ± 102 mm^2^), Grewal [20] (490 ± 220 μm and 0.320 ± 0.120 mm^2^) and Tan [21] (486 ± 36 μm and 0.173 ± 0.014 mm^2^), respectively.

We observed the excellent reproducibility of angle measurements made using the RTVue OCT. Similarly, good reproducibility was detected by Tan et al. [21] for the Visante OCT in Asian subjects and by Römkens et al. [22] for the CASIA OCT in Caucasians, in both cases whether the exam was performed by experts or non experts.

Among the main subject factors examined in our study, women and hyperopes showed a narrower angle width (*p* < 0.001), in agreement with the findings of other authors [10–12, 15].

Age in our large population of subjects examined, showed negative univariate correlation with TIA. This observation is consistent with those of other authors, [10–12] and in particular with the findings of Sun et al. [23] who noted that AOD_500_ also diminishes with age obtaining a similar correlation (R = -0.462; *p* < 0.005) to the value recorded in our study (R = -0.484; *p* < 0.001). However, the multivariate model identified age as a weak predictor of angle width, as also reported by Lavanya et al. and Foo et al. [15, 16, 24].

In the present sample of healthy eyes, no correlation was detected between angle width and IOP even when we only considered narrow angles, in agreement with the findings of Amerasinghe et al. [12]. In contrast, Chong et al. [25] argued that the extent of angular closure could be correlated with IOP. However, the difference detected was only a 1.3 mmHg increase for angles showing closure in all 4 quadrants compared to no quadrant.

In the eyes examined here, strong correlation was detected between angle width and the anterior chamber variables, ACV and ACD (R ≥ 0.818; *p* < 0.001) such that narrower chambers showed a narrower angle [10, 12, 15, 18, 23]. Correlation with WTW was much weaker (R = 0.239; *p* < 0.001). Iris thickness also showed weak correlation with the angle measurements though some authors have argued that this factor is a significant determinant of angle width [18, 24].

The multivariate model constructed in our study for Caucasian subjects based on six variables was able to explain 76.4 % of the final variation observed in angle width. The other large model described to date by Foo et al. [16] for Asian subjects included six anterior chamber variables measured by OCT: ACV and ACD, ACW or horizontal scleral spur-to-spur distance, lens vault, iris surface area and iris thickness. This model served to explain 85.5 % of the variation produced in AOD_750_. However, it should be mentioned that this model failed to consider factors as important as spherical error or sex.

Among the main limitations of our study was the fact that only the horizontal quadrants were studied. This was because of the poor quality of images obtained for the superior and inferior quadrants and the need to manipulate the eyelids. Another limitation was that we did not measure the lens vault despite the known contribution of this factor to angle width variation [18, 23, 24].

To the best of our knowledge, this is the first study to examine the use of FD-OCT to assess the iridocorneal angle in a large population of healthy Caucasian subjects. Our study offers reference values of angle width and demographic and ocular variable correlates for Caucasian subjects. We speculate that this technique will also help understand the anatomical changes that occur in glaucoma surgery, especially in angle surgery [26, 27].

## Conclusions

In conclusion, our findings indicate that FD-OCT is a reliable examination technique to objectively assess the anterior chamber angle. Using this device in a large number of healthy Caucasians, strong correlation was observed between angle measurements and ACV, ACD, spherical refractive error and sex, and no correlation was detected with IOP. ACV emerged as the main determinant of anterior angle width.

### Ethics and consent to participate

Signed informed consent was obtained from subjects meeting the study’s inclusion and exclusion criteria. The study protocol adhered to the tenets of the Declaration of Helsinki and received Institutional Review Board approval from the Centro Internacional de Oftalmología Avanzada, Madrid (Spain).

### Consent to publish

Not applicable

### Availability of data and materials

All the data supporting our findings is contained within the manuscript.

## Abbreviations

AOD_500_: angle opening distance 500 μm from the scleral spur
FD-OCT: Fourier domain optical coherence tomography
TIA: trabecular-iris angle
TISA_500_: trabecular-iris space area 500 μm from the scleral spur
